# Removal

**Published:** 1862-10

**Authors:** 


### REMOVAL.

The old Toland dental depot establishment of 38 West 4th st. has been removed to corner of 4th and Walnut sts., in Carlisle's Building, to a room much more commodious in every respect; it is large and well lighted; this, as all know, is a very important matter in the selection of teeth, and indeed in the selection of all dental goods. In addition to goodness of the place, there is a large and well arranged stock from which to select. T.

